# Industrial robot selection using a multiple criteria group decision making method with individual preferences

**DOI:** 10.1371/journal.pone.0259354

**Published:** 2021-12-16

**Authors:** Jinling Zhao, Yubing Sui, Yang Xu, K. K. Lai

**Affiliations:** 1 School of Economics, Shen Zhen Polytechnic, Shenzhen, China; 2 School of Finance and Economics, Shenzhen Institute of Information Technology, Shenzhen, China; 3 School of Economics and Management, Xi’an Technological University, Xi’an, China; 4 Shenzhen Institute, University of International Business and Economics, Shenzhen, China; University of Defence in Belgrade, SERBIA

## Abstract

This paper proposes a multiple criteria group decision making with individual preferences (MCGDM-IP) to address the robot selection problem (RSP). Four objective criteria elicitation approaches, namely, Shannon entropy approach, CRITIC approach, distance-based approach, and ideal-point approach, are proposed to indicate individual decision makers. A preliminary group decision matrix is therefore formulated. Both preferential differences representing the preference degrees among different robots, and preferential priorities representing the favorite ranking of robots for each individual decision maker, are analyzed to propose a revised group decision matrix. A satisfaction index is developed to manifest the merits of the proposed MCGDM-IP. An illustrative example using the data drawn from previous literature is conducted to indicate the effectiveness and validity of MCGDM-IP. The results demonstrate that the MCGDM-IP could generate a more satisfactory scheme to evaluate and select industrial robots, with an improvement of group satisfactory level as 2.12%.

## 1. Introduction

The worldwide manufacturing industry is confronting with great challenges such as changing trends in consumer choices, aging society, shortage of resources and skilled workers, and demand for local pro- ductions. The solutions to these challenges could be delivered by using flexible industrial robot-based automation. Starting from 2010, the demand for industrial robots has been expanded considerably because of the continued technical innovation in industrial robots and ongoing trend toward automation. In 2018, global robot installations increase by 6% to 422,271 units, worth USD 16.5 billion [1]. As the crucial part of intelligentization and digitalization of the manufacturing industry, industrial robots are programmable, automated, and capable of movement on three or more axes. Specific applications of robots include assembly, disassembly, welding, painting, pick and place for printed circuit boards, packaging and labeling, palletizing, product inspection, and testing; all applications accomplished with high speed, endurance, and precision. Because there are a vast amount of industrial robots with miscellaneous features, capabilities, and specifications in the market, the decision maker in particular needs to select the most appropriate robots in consideration of both financial and technical factors, for the purpose of enabling manufacturing enterprises to produce high-quality products in a cost-effective manner [2]. This indicates that the multiple criteria decision making (MCDM) could be an effective instrument to solve the robot selection problem (RSP). Koulouriotis and Ketipi present an elaborative, extensive, and aggregate review of various MCDM methods for addressing the RSP, and perform a comparative analysis among them, respectively [3,4].

Robot selection usually spends a large capital expenditure and entails knowledge from several persons with diverse functional backgrounds within a company. Therefore, the robot selection decision is typically made by a committee or a group of experts, instead of by a single person [5,6] Such groups might be composed of top management teams, board of directors, and/or financial officers. Individual decisions are usually featured with a high level of variability, which naturally occurs when the decision making entity is made up of several individuals, as in the case of decision making by management teams [7]. Therefore, the problem boils down to how one could combine the individual opinions to realize a preferred decision [8] Since different individuals may often have their own views/judgments, how individual opinions should be integrated as a group-level decision has meaningful insights for organizational decision and the related performance [9]. However, choosing the appropriate method to aggregate opinions is not an easy task, as there are various aggregation schemes and which one is the best may be contingent upon many elements.

Recently, the family of multiple criteria group decision making (MCGDM) methods has received substantial attentions in solving RSP. Specifically, the MCGDM involves a set of feasible alternatives that are evaluated based on multiple, conflicting and non-commensurate criteria by a group of individuals. Choudhury et al. [10] develop a multi-agent system (MAS) based negotiation model to address an advanced technology selection problem with multi-person, multi-criteria and multi-preference. Ja- ganathan et al. [11] give an integrated fuzzy AHP based group decision support system to facilitate the evaluation and selection of new manufacturing technologies in the presence of intangible attributes and uncertainty. Chuu [12,13] proposes a MCGDM model using fuzzy multiple attributes analysis to assess the suitability of manufacturing technology. A new fusion method of fuzzy information is proposed to manage information evaluated in different linguistic scales (multi-granularity linguistic term sets) and numerical scales. Rashid et al. [14] devise a method to aggregate the opinions of several decision makers on different criteria, regarding a set of alternatives, where the judgment of the decision makers are represented by generalized interval-valued trapezoidal fuzzy numbers. Keshavarz Ghorabaee M [15] presents a MCGDM approach for robot selection in the context of type-2 fuzzy sets, in which a method based on VIKOR with interval type-2 fuzzy numbers is developed. The stability of proposed approach is analyzed by using seven sets of criteria weights and the Spearman correlation coefficient. Fu et al. [16] identify various decision makers (DMs) by different weight determination methods, and propose to use the stochastic multicriteria acceptability analysis for group decision making (SMAA- 2) to facilitate the industrial robot evaluation and selection. Ali and Rashid employ a group best-worst method for robot selection [17].

Almost all of the existing RSP MCGDM models in particular follow the rationale that the group opinions for an alternative should be a weighted sum of the individual member’s opinions for the alternatives, and the essence of MCGDM is therefore to elicit the weights based upon the interpersonal comparison of opinions and upon the power or relative importance of each individual in the group [18]. In this sense, the individual decision makers in the group expect to have better commitments and thereby further implement the consentaneous decision [19]. However, in the case that some DMs have specific preferences about certain alternatives, the extant RSP MCGDM models fail to characterize the group preferences among alternatives from the practical viewpoint. This could give rise to a dilemma when some DMs have extreme preferences about certain alternatives, the group opinion aggregated by the weighted sum scheme might not precisely reveal their substantial attention to the problem, and consequently result in a scenario with poor commitments from group members. The research questions of this work are summarized as follows. First, what is the MCGDM framework for the RSP? Second, how to analyze the preference structure of the RSP? Third, how to measure the effectiveness of our method? In this regard, the primary goal of this work is to propose a MCGDM method with individual preferences (MCGDM-IP) to effectively and rationally aggregate individual opinions in consideration of their corresponding individual preferences. Furthermore, in accordance with Huang et al. [20] and Fu et al. [21], this paper explores the individual preferences from the aspects of both the preferential differences and preferential priorities for individual DMs’ assessments of alternatives in a decision group. Specifically, the preferential differences indicate the preference degrees among different alternatives, and the preferential priorities denote the favorite ranking of the alternatives for each individual DM.

In general, the proposed MCGDM-IP for industrial robot selection is proceeded in three stages. First, different individual DMs are identified by proposing several weight elicitation methods that determine weights corresponding to robot selection criteria. The developed procedures, however, tend to generate different weights for the same RSP. On the other hand, in a RSP with multiple DMs, achieving consensus about exact weights might not be easy [22]. This in a sense reveals the preference heterogeneities among different DMs. A decision group with a preliminary decision matrix is therefore formulated, the elements of which are the performance results of alternatives evaluated by individual DMs. Second, the preferential differences among alternatives and preferential priorities of individual DMs are analyzed to produce a revised decision matrix, according to which the final group opinion is derived by aggregating the individual opinions to evaluate and select the robots. Third, a satisfaction index that reflects the satisfaction status of a group decision associated with each individual DM is constructed to manifest the merits of MCGDM-IP.

In comparison with the existing MCGDM models for industrial robot selection, the proposed MCGDM-IP has the following prominent features. First, each individual DM objectively derives the criteria weights based on the evaluation dataset itself. This can effectively reduce the decision bias and to some extent improve the decision quality. Second, the preferential differences among various robots and preferential priorities for each individual DM are analyzed to obtain a revised group decision matrix, and thereby to enhance the commitment among group members. Third, the satisfaction status of individual DMs and group are separately and comprehensively measured to demonstrate the feasibility and superiority of MCGDM-IP.

The rest of this study is organized as below. In Section 2, we propose the MCGDM-IP for resolving the RSP, followed by a numerical illustration in Section 3. This study is concluded in Section 4 by discussing the limitations of proposed method and suggestions for future research.

## 2. Method

Assume that *m* robots are assessed and selected in terms of *n* criteria. Each criterion can be either subjective or objective. Subjective criteria are commonly qualitative in nature, and could be converted to numerical values by linguistic modeling schemes, and objective criteria could be numerically described and measured, such as load capacity, velocity, repeatability, and cost, among others. Let *x*_*ij*_, *i* = 1,2,…,*m*, *j* = 1,2,…,*n* represent the performance of robot *i* with respect to criterion *j*. In accordance with the observation that robot selection decision is typically made by a group of individual DMs, this study proposes a novel MCGDM-IP to address the RSP. The general framework of MCGDM-IP is presented as below.

**Fig 1:**
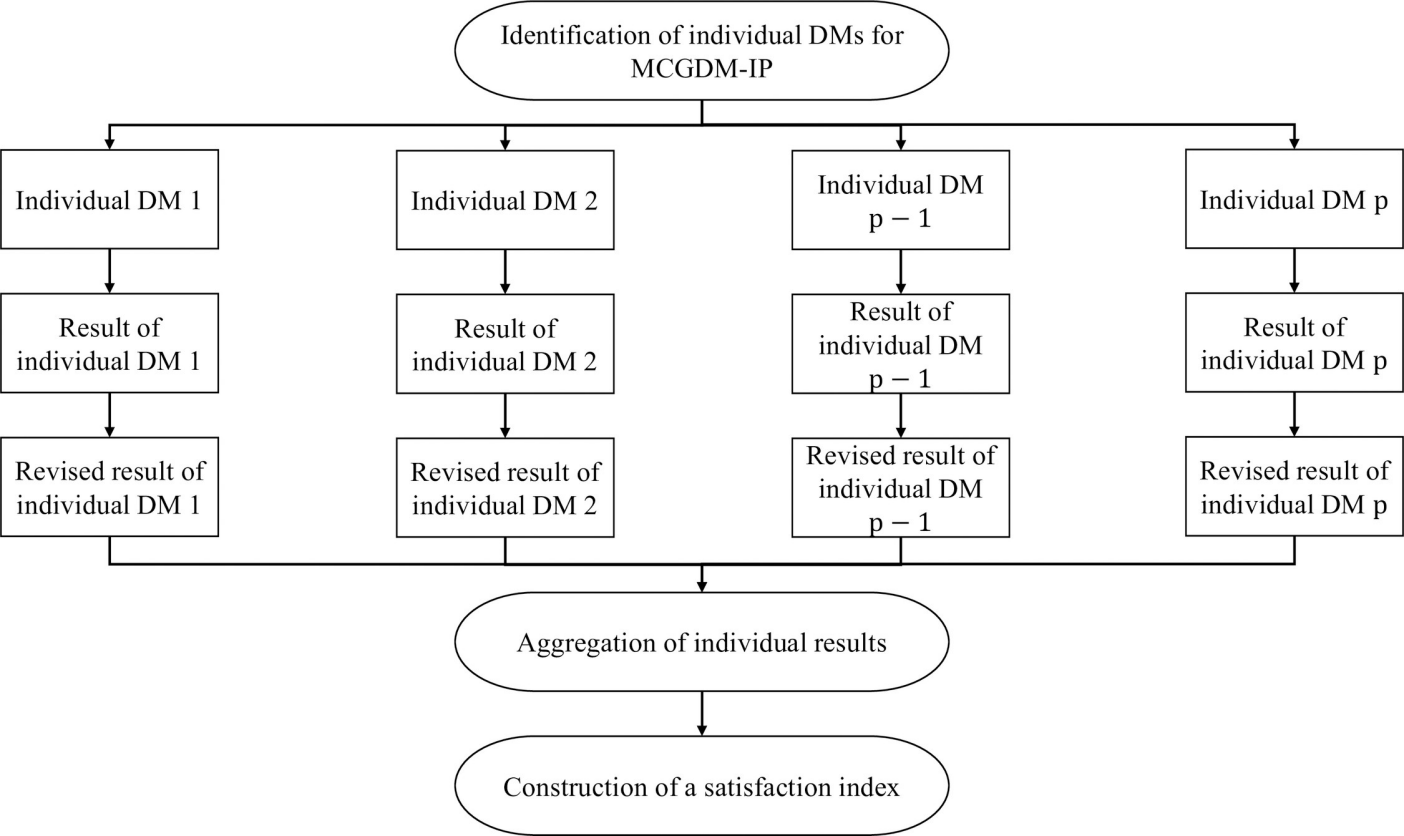
The general framework of MCGDM-IP.

According to Fig 1, the proposed MCGDM-IP in general is composed of three main stages: identification of individual DMs and implementation of standard criteria weight elicitation process for each individual DM, aggregation of individual opinions with preferential differences and preferential priorities, and construction of a satisfaction index, which are explicitly elaborated in the following subsections.

### 2.1 Individual decision making

A variety of MCDM methods employ weights to describe the relative significance of different criteria. Many weight determination methods have been proposed for finding the actual weights of a DM [22]. Often different DMs may suggest different criteria weights for the same problem. Similar to Yu and Lai [23] and Fu et al. [16], the individual DMs are reasonably represented by different criteria weight determination approaches in this paper. For robot selection decision, an individual DM may represent a department such as Production or Finance. The individual DM would presumably reflect the preferences of the department. Production, for example, may believe that the performance characteristics of the candidate robots, such as velocity and programming flexibility, are more important, but Finance may believe price or cost is a more important criterion.

In this paper, we propose to identify individual DMs using several objective criteria weight determination approaches, namely, Shannon entropy approach, CRITIC approach, distance-based approach, and ideal-point approach [16]. The main advantage of these objective approaches is the reduction of decision bias by means of ignoring the subjective judgments of the individual DMs [24]. Objective criteria weight elicitation approaches are usually applicable when individual DMs disagree on the precise values of criteria weights [23] More specifically, the rationale behind objective criteria weight elicitation approaches is that the importance level of a criterion is a function of the information conveyed by this criterion, relative to a whole set of alternatives. For the sake of alleviating the negative effect of data magnitude and making the comparison across different individual DMs feasible, *x*_*ij*_, *i* = 1,2,…,*m*, *j* = 1,2,…,*n* are normalized by means of

{yij=xij∑i=1mxij,forbenefit−typecriteria;yij=1xij∑i=1m1xijforcost−typecriteria; (1)

Individual DM *k* determines the weight associated with each criterion $w_{j}^{k}$, *j* = 1,2,…,*n* to maximize the overall performance of robot *i*, *i* = 1,2,…,*m*;

$$v_{ik}=\sum_{j=1}^{n}y_{ij}w_{j}^{k},\;i=1,2,\ldots ,m$$ (2)

In line with the observation that robot selection decision is particularly made by a management committee within a company, and different individual DMs usually elicit different weights for the same problem, this study adopts the following four objective criteria weight determination approaches that reveal problem complexity and decision responsibility.

#### 2.1.1 Shannon entropy approach

Shannon entropy approach has been extensively employed and integrated (for example, the combination of the CoCoCo and the Shannon entropy to elicit criteria weights in MCDM literature [25]. The rationale of Shannon entropy approach is that the larger the degree of diversity within a criterion dataset, the higher the weight with respect to that criterion. In other words, the smaller the entropy associated with a criterion, the larger the discriminating power of that criterion in ranking alternatives. Therefore, the working process of Shannon entropy approach that determine the criteria weights is introduced below:

- Normalizing the input data using (1).
- Computing the Shannon entropy for each criterion *j*, $e_{j}=-[\ln{\left(m\right)}^{-1}]\sum_{i=1}^{n}y_{ij}\ln(y_{ij})$, *j* = 1,2,…,*n*.
- Calculating the degree of discriminability for each criterion *j*, *d*_*j*_ = 1−*e*_*j*_, *j* = 1,2,…,*n*.
- Deriving the criteria weights with respect to each criterion *j*, $w_{j}=\frac{d_{j}}{\sum_{j=1}^{n}d_{j}}$, *j* = 1,2,…,*n*.
- Optimizing the overall performance of robots using (2).

#### 2.1.2. CRITIC approach

The CRITIC approach elicits objective criteria weights based on the quantification of two fundamental notions of MCDM: the contrast intensity and the conflicting character of the evaluation criteria [26], which is further modified by Mališa Žižović [27]. The working procedure of CRITIC approach is presented as follows:

- Normalizing the input data using (1).
- Computing the standard deviation for criterion *j*, *σ*_*j*_, which quantifies the contrast intensity of the related criterion. In this regard, *σ*_*j*_ could be recognized as a measure of the value of criterion *j* to the decision making process.
- Constructing a symmetric matrix, with *n*×*n* dimension and a generic element *r*_*jk*_. Specifically, *r*_*jk*_ is the linear correlation coefficient between criteria *j* and *k*. The more discordant of the scores of the alternatives in criteria *j* and *k*, the smaller the value *r*_*jk*_. Therefore, $\sum_{k=1}^{n}\left(1-r_{jk}\right)$, represents a measure of the conflict caused by criterion *j* with respect to the decision situation involving the rest of criteria.
- Calculating the amount of information. Information embedded in MCDM problems consists of contrast intensity and conflict of the decision criteria. The amount of information *I*_*j*_ thus could be decided by quantifying two expressions using a multiplicative aggregation formula: $I_{j}=\sigma_{j}•\sum_{k=1}^{n}\left(1-r_{jk}\right)$.
- Determining the criteria weights with respect to each criterion *j*, $w_{j}=\frac{I_{j}}{\sum_{j=1}^{n}I_{j}}$, *j* = 1,2,…,*n*. The large the value *I*_*j*_, the more information reflected by the corresponding criterion *j* and the higher its relative importance for the decision making process.
- Optimizing the overall performance of robots using (2).

#### 2.1.3 Distance-based approach

The mechanism of distance-based approach is to minimize the discrepancy between self- and peer- evaluation outcomes. This discrepancy could be understood as the information redundancy or noisy generated in the evaluation procedure, which will be reduced or eliminated to produce reasonable evaluation outcomes Fu et al [28]. In this sense, the smaller the discrepancy, the more consistent between self- and peer-assessment outcomes, the better the assessment approach. *y*_*ij*_ indicates the performance of alternative *i* with respect to criterion *j*, and is therefore defined as the self-evaluation outcome. From the veiwpoint of alternative *i*, the arithmetic average of all *y*_*ij*_, namely ${\bar{y}}_{i}=\frac{1}{n}\sum_{j=1}^{n}y_{ij}$, *i* = 1,2,…,*m*, is regarded as the peer-evaluation outcome.

The discrepancy between self- and peer-evaluation outcomes could be depicted by the Euclidean distance function. Naturally, each alternative seeks to minimize the corresponding discrepancy as:

$$f_{i}=\min\sum_{j=1}^{n}\left[{\left(y_{ij}-{\bar{y}}_{i}\right)}^{2}w_{j}^{2}\right]$$ (3)

$$s.t.\sum_{j=1}^{n}w_{j}=1$$ (4)

From the systematic viewpoint, a multiple-objective programming model is provided to optimize the performance results of all alternatives:

$$\{\begin{array}{c} \{\begin{array}{c} f_{1}=\min\sum_{j=1}^{n}\left[{\left(y_{1j}-{\bar{y}}_{1}\right)}^{2}w_{j}^{2}\right]\\ f_{2}=\min\sum_{j=1}^{n}\left[{\left(y_{2j}-{\bar{y}}_{2}\right)}^{2}w_{j}^{2}\right]\\ \vdots \\ f_{m}=\min\sum_{j=1}^{n}\left[{\left(y_{mj}-{\bar{y}}_{m}\right)}^{2}w_{j}^{2}\right] \end{array}\\ s.t.\sum_{j=1}^{n}w_{j}=1 \end{array}$$ (5)

A single-objective programming model is then proposed using linear equal weighted summation method:

$$\{\begin{array}{c} F=\min\sum_{i=1}^{m}\sum_{j=1}^{n}\left[{\left(y_{ij}-{\bar{y}}_{i}\right)}^{2}w_{j}^{2}\right]\\ s.t.\sum_{j=1}^{n}w_{j}=1 \end{array}$$ (6)

A Lagrange function is constructed to solve the quadratic programming model (6):

$$L=\min\sum_{i=1}^{m}\sum_{j=1}^{n}\left[{\left(y_{ij}-{\bar{y}}_{i}\right)}^{2}w_{j}^{2}\right]+\lambda (\sum_{j=1}^{n}w_{j}-1)$$ (7)

The Hessian matrix of *L* with respect to *w*_*j*_ is

$$H=[\begin{array}{cccc} \frac{\partial^{2}L}{\partial w_{1}^{2}} & \frac{\partial^{2}L}{\partial w_{1}\partial w_{2}} & \cdots & \frac{\partial^{2}L}{\partial w_{1}\partial w_{n}}\\ \frac{\partial^{2}L}{\partial w_{2}\partial w_{1}} & \frac{\partial^{2}L}{\partial w_{2}^{2}} & \cdots & \frac{\partial^{2}L}{\partial w_{2}\partial w_{n}}\\ \vdots & \vdots & \vdots & \vdots \\ \frac{\partial^{2}L}{\partial w_{n}\partial w_{1}} & \frac{\partial^{2}L}{\partial w_{n}\partial w_{2}} & \cdots & \frac{\partial^{2}L}{\partial w_{n}^{2}} \end{array}],$$

in which the diagonal elements are positive, and the others are zero. Therefore, the Hessian theorem indicates that the Lagrange function *L* has a minimum value. By solving $\{\begin{array}{c} \frac{\partial L}{\partial \lambda }=0\\ \frac{\partial L}{\partial w_{j}}=0 \end{array}$, we obtain the optimal solutions to the single-objective programming model (6) as:

$$w_{j}=\frac{1}{\sum_{j=1}^{n}{\left[\sum_{i=1}^{m}{\left(y_{ij}-{\bar{y}}_{i}\right)}^{2}\right]}^{-1}\times \sum_{i=1}^{m}{\left(y_{ij}-{\bar{y}}_{i}\right)}^{2}}$$ (8)

Then the overall performance of robots is optimized using (2).

#### 2.1.4 Ideal-point approach

The ideal-point approach attempts to make all alternatives as close to the ideal point as possible [24]. This approach is intuitively appealing because all alternatives always seek to maximize their performance and therefore to be selected. Similar to Ma et al. [24], we construct a weighted decision matrix [*Y*_*ij*_]_*m*×*n*_, where *Y*_*ij*_ = *w*_*j*_*y*_*ij*_. Therefore, the ideal-point is defined as $Y^{*}=\{Y_{1}^{*},Y_{2}^{*},\ldots ,Y_{n}^{*}\}$, in which

$$Y_{j}^{*}=\underset{i}{\max}\{Y_{ij}\}=\underset{i}{\max}\{w_{j}y_{ij}\}=\underset{i}{\max}\{y_{ij}\}w_{j}=y_{j}^{*}w_{j}$$ (9)

and $y_{j}^{*}=\underset{i}{\max}\{y_{ij}\}$ is the ideal value associated with criterion *j*. The ideal-point could be either virtual or real and is in particular regarded as the target to achieve.

The difference between alternative *i* and ideal-point could be quantified by using the Euclidean distance function below:

$$D_{i}=\sum_{j=1}^{n}{\left(Y_{ij}-Y_{j}^{*}\right)}^{2}$$ (10)

$$\begin{array}{cc} & = \end{array}\sum_{j=1}^{n}\left[{\left(y_{ij}-y_{j}^{*}\right)}^{2}w_{j}^{2}\right]$$ (11)

From a systematic standpoint, a multiple-objective programming model is proposed to optimize the performance of all alternatives:

$$\{\begin{array}{c} \{\begin{array}{c} g_{1}=\min\sum_{j=1}^{n}\left[{\left(y_{1j}-y_{j}^{*}\right)}^{2}w_{j}^{2}\right]\\ g_{2}=\min\sum_{j=1}^{n}\left[{\left(y_{2j}-y_{j}^{*}\right)}^{2}w_{j}^{2}\right]\\ \vdots \\ g_{m}=\min\sum_{j=1}^{n}\left[{\left(y_{mj}-y_{j}^{*}\right)}^{2}w_{j}^{2}\right] \end{array}\\ s.t.\sum_{j=1}^{n}w_{j}=1 \end{array}$$ (12)

Performing the analogous process of distance-based approach, the optimal solutions to model (12) are derived as:

$$w_{j}=\frac{1}{\sum_{j=1}^{n}{\left[\sum_{i=1}^{m}{\left(y_{ij}-y_{j}^{*}\right)}^{2}\right]}^{-1}\times \sum_{i=1}^{m}{\left(y_{ij}-y_{j}^{*}\right)}^{2}}$$ (13)

Then the overall performance of robots is optimized using (2).

### 2.2 Aggregation of individual opinions with individual preferences

Consider a general scenario with m alternatives being assessed by *p* DMs, and *v*_*ik*_, *i* = 1,2,…,*m* represent the performance results of alternative *i*, *i* = 1,2,…,*m* evaluated by individual DM *k*, *k* = 1,2,…,*p*. In the presence of different criteria weights determined by different individual DMs, there exist various evaluation results for alternatives. An *m*×*p* preference decision matrix is therefore proposed as follows:

$$V_{m\times p}=[\begin{array}{ccccc} v_{11} & \cdots & v_{1k} & \cdots & v_{1p}\\ \vdots & \vdots & \vdots & \vdots & \vdots \\ v_{21} & \cdots & v_{ik} & \cdots & v_{ip}\\ \vdots & \vdots & \vdots & \vdots & \vdots \\ v_{m1} & \cdots & v_{mk} & \cdots & v_{mp} \end{array}]$$ (14)

In line with Huang et al [20], we analyze the individual preferences from the standpoints of preferential differences and preferential priorities to enhance the commitments among group members.

Because the second moment (variance or standard deviation) reflects the dispersion of a sample in statistics, we employ the second moment of the performance results evaluated by an individual DM for all robots, to measure the dispersion of an individual DM’s preferences. We therefore calculate the standard deviation with respect to the individual DM *k*, *k* = 1,2,…,*p* as below:

$$\sigma_{k}=\sqrt{\frac{\sum_{i=1}^{m}{\left(v_{ik}-{\bar{v}}_{k}\right)}^{2}}{m-1}},\;k=1,2,\ldots ,p$$ (15)

in which ${\bar{v}}_{k}$ denotes the mean of the performance outcomes of all robots assessed by individual DM *k*. Since each individual DM has her/his own characteristic preference distribution among different robots, *σ*_*k*_ is thus employed to evaluate the dispersion degree of their preferences on all robots. A larger *σ*_*k*_ indicates that individual DM *k* has a better capability to decisively distinguish her/his preferences among different robots. In this regard, considering various decisiveness of different individual DMs in a group, the performance outcomes evaluated by each individual DM should be adjusted for aggregation. According to Huang et al [20]. *σ*_*k*_ could be recognized as the second moment adjusting factor of preferential difference for individual DM *k*, and is therefore utilized as a power term to adjust *v*_*ik*_, *i* = 1,2,…,*m*, *k* = 1,2,…,*p*. Such an adjustment is aimed at extending the range of *v*_*ik*_ with larger preferential difference to enhance the influence of such an individual DM on the final decision. The revised expression of *v*_*ik*_ is:

vik(1)={vik1−σkifvik≥v¯kvik1(1−σk)ifvik<v¯k (16)

where the performance outcome larger than the average would turn into even larger, and the performance outcome smaller than the average would turn into even smaller. This impact would increase as the preferential difference increases.

In addition, individual DMs may sometimes manifest strong unwillingness or willingness for her/his relatively unfavorable or favorable robots. Since the third moment measures the skewness of a sample in statistics, it is employed to quantify the strength of an individual DM’s willingness to adopt favorable robots, which is presented as:

$$\gamma_{k}=\sqrt[{3}]{\frac{m}{\left(m-1)(m-2\right)}(\sum_{i=1}^{m}{\left(\frac{v_{ik}-{\bar{v}}_{k}}{\sigma_{k}}\right)}^{3})}\;k=1,2,\ldots ,p$$ (17)

The preference aggregation in a decision group may be influenced by the skewness of preferences among robots for an individual DM. An individual DM with a left-skewed pattern of performance outcomes among robots gives high performance outcomes to most robots and low performance out- comes to a relatively small proportion of robots. This indicates that this individual DM may have similar preferences for most robots that she/he favors, but would be extremely uncomfortable if any of those robots that she/he does not favor is adopted. Since the third moment could be either positive or negative, we make an adjustment to meet the requirements in aggregation process. Specifically, if the third moments for all individual DMs are positive, they could be standardized as $\gamma_{k}^{NOR}=\frac{\gamma_{k}}{\sum_{k=1}^{p}\gamma_{k}}$. Otherwise, if there is one or more negative third moments, we define $\gamma_{k}=\underset{l}{\min}\{\gamma_{l}\}$, for all *γ*_*l*_<0, $\gamma_{k}^{\prime }=|\gamma_{k}|$, and $\gamma_{t}^{\prime }=\gamma_{t}+(\gamma_{k}^{\prime }-\gamma_{k})$, for all *t*≠*k*, then the third moments could be standardized as $\gamma_{k}^{NOR}=\frac{\gamma_{k}^{\prime }}{\sum_{k=1}^{p}\gamma_{k}^{\prime }}$. According to Huang et al [20], the cumulative distribution function (CDF) of an exponential distribution is employed to formulate the adjustment that combines the second and third moments for preferential differences. Therefore, the revised group decision matrix is depicted as:

$$v_{ik}^{\left(2\right)}=\{\begin{array}{cc} v_{ik}^{\left(1\right)}+(1-v_{ik}^{\left(1\right)})\gamma_{k}^{NOR}[1-\exp(-v_{ik}*{\bar{v}}_{k})], & \mathrm{if}\;v_{ik}\geq {\bar{v}}_{k}\\ v_{ik}^{\left(1\right)}-v_{ik}^{\left(1\right)}\gamma_{k}^{NOR}[1-\exp(-v_{ik}*{\bar{v}}_{k})], & if\;v_{ik}<{\bar{v}}_{k} \end{array}$$ (18)

Next, we untangle the preferential priorities in the group decision matrix. Individual DMs are particulary aware of whether their most preferred nation is adopted, and then the second preferred, the third, and so on. This psychological effect may influence the group decision process and should be considered with respect to the compromise among individual DMs. Furthermore, it is also significant to consider the plurality of votes. The preferential priorities of robot i are defined as: $\delta_{i}=\frac{\sum_{k=1}^{p}\theta_{ik}}{\sum_{i=1}^{m}\sum_{k=1}^{p}\theta_{ik}}$, in which $\theta_{ik}=\frac{m}{\phi_{ik}}$, and *θ*_*ik*_ and *ϕ*_*ik*_ represent the priority coefficient and preferential ranking of robot *i* for individual DM *k*, respectively. By doing so, the preferential priorities of the robots with higher ranking will be exponentially increasing.

Ultimately, the performance outcomes considering both preferential differences and preferential priorities are modified as:

$$v_{ik}^{\left(3\right)}=\{\begin{array}{cc} v_{ik}^{\left(1\right)}+(1-v_{ik}^{\left(1\right)})\delta_{i}\gamma_{k}^{NOR}[1-\exp(-v_{ik}*{\bar{v}}_{k})], & \mathrm{if}\;v_{ik}\geq {\bar{v}}_{k}\\ v_{ik}^{\left(1\right)}(1-\delta_{i})\gamma_{k}^{NOR}[1-\exp(-v_{ik}*{\bar{v}}_{k})], & if\;v_{ik}<{\bar{v}}_{k} \end{array}$$ (19)

In this regard, the preference decision matrix *V*_*m*×*p*_ is consequently revised as ${\left[v_{ik}^{\left(3\right)}\right]}_{m\times p}$. In accordance with the “same decision power” assumption in group decision systems proposed by Huang et al [20], the discriminative performance of robot i is thus obtained by taking the arithmetic mean value of its performance results across the p individual DMs:

$$v_{i}=\frac{\sum_{k=1}^{p}v_{ik}^{\left(3\right)}}{p},\;i=1,2,\ldots ,m$$ (20)

which are capable of capturing the preferential differences among various robots and preferential priorities for each individual DM.

### 2.3 A satisfaction index

To justify the effectiveness of our MCGDM-IP, this study proposes a satisfaction index to measure and compare the degrees of satisfaction between MCGDM-IP (*v*_*i*_) and individual DM (*v*_*ik*_). The difference between the performance results of a robot evaluated by decision group and an individual DM is an appropriate yardstick to measure the satisfaction degree, and is thus defined as below:

$$\varphi_{ik}=|v_{ik}-v_{i}|.$$ (21)

The mentioned satisfactory difference could be normalized as $\psi_{ik}^{NOR}=\frac{\varphi_{ik}}{\sum_{k=1}^{p}\varphi_{ik}}$, which has a conceptual understanding as the distance. In other words, the larger the satisfactory difference, the more unsatisfactory the alternative.

In addition to the satisfactory difference, the differences between the rankings derived according to vi and any of other robots may also have a notable influence on the satisfactory levels of robots. Similarly, the difference of preferential rankings is defined as

$$\varsigma_{ik}=|\rho_{ik}-\rho_{i}|$$ (22)

where *ρ*_*ik*_ denotes the preferential ranking of robot *i* assessed by individual DM *k*, and *ρ*_*i*_ represents the preferential ranking of robot *i* with respect to *v*_*i*_.

Finally, according to Siskos et al [29], the above two satisfactory aspects could be integrated to build the satisfaction index for individual DM *k* as:

$$\pi_{k}=1-\frac{1}{m}\sum_{k=1}^{p}\left[{\left(\psi_{ik}^{NOR}\right)}^{\varsigma_{ik}}v_{i}\right]$$ (23)

In line with the exiting literature [19,20,30], the arithmetic average of *π*_*k*_, *k* = 1,2,…,*p* is utilized to indicate the comprehensive satisfactory levels for all individual DMs

## 3. Numerical illustration

In this section, the proposed MCGDM-IP is applied to solve a RSP with the consideration of five criteria: Cost, Handling coefficient, Load capacity, 1/Repeatability and Velocity:

- cost, i.e., the robot’s catalogue price;
- handling coefficient, i.e., diameter, elevation, basic rotation, roll, pitch, and yaw;
- load capacity, i.e., the robot’s maximum transportable weight;
- repeatability, i.e., the measure of the accuracy with which the robot permits the end effector to return to a specific point;
- velocity, i.e., the end effector’s maximum attainable speed.

This case has been extensively investigated using various Data Envelopment Analysis (DEA) models [31–33]. The input data for robot selection are listed in the Table 1 below.

**Table 1:** Input data for robot selection.

Robot	Cost (USD)	Handling coefficient	Load capacity (kg)	1/Repeatability (mm)	Velocity (m/s)
R1	100,000	0.995	85	1.7	3.00
R2	75,000	0.933	45	2.5	3.60
R3	56,250	0.875	18	5.0	2.20
R4	28,125	0.409	16	1.7	1.50
R5	46,875	0.818	20	5.0	1.10
R6	78,125	0.664	60	2.5	1.35
R7	87,500	0.880	90	2.0	1.40
R8	56,250	0.633	10	8.0	2.50
R9	56,250	0.653	25	4.0	2.50
R10	87,500	0.747	100	2.0	2.50
R11	68,750	0.880	100	4.0	1.50
R12	43,750	0.633	70	5.0	3.00

**Fig 2:**
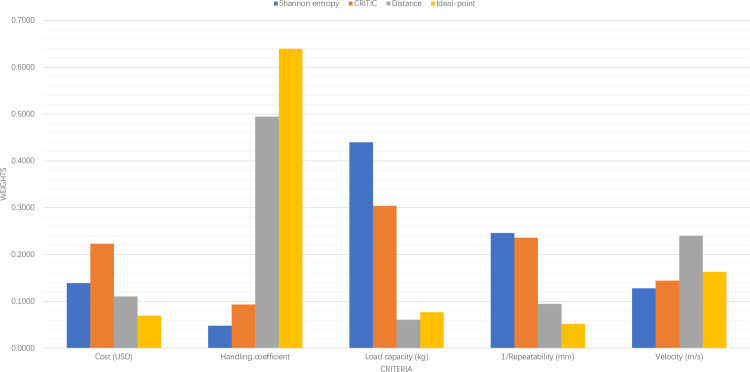
Criteria weights.

In this work, four individual DMs are identified using Shannon entropy approach, CRITIC approach, distance-based approach, and ideal-point approach. The criteria weights are reported and compared in Table 2 and Fig 2 below. Both Shannon entropy approach and CRITIC approach assign the largest and smallest weights to Load capacity and Handling coefficient, respectively. Interestingly, both distance-based approach and ideal-point approach consider Handling coefficient as the most important criterion, and Load capacity and Repeatability as the least important criteria, respectively. These discrepancies among criteria weights could be caused by different knowledge and expectations of individual DMs, and thus make the implementation of MCRSP extremely difficult.

**Table 2:** Criteria weights.

Individual DM	Cost (USD)	Handling coefficient	Load capacity (kg)	1/Repeatability (mm)	Velocity (m/s)
Shannon entropy	0.1389	0.0479	0.4396	0.2458	0.1277
CRITIC	0.2229	0.0931	0.3039	0.2360	0.1441
Distance	0.1105	0.4944	0.0608	0.0944	0.2399
Ideal-point	0.0694	0.6393	0.0765	0.0518	0.1629

By means of the obtained criteria weights, the robot performance results evaluated by four individual DMs are presented in Table 3 below. It is observed that Shannon entropy approach, CRITIC approach, distance-based approach, and ideal-point approach rank R11, R12, R2, and R1 at the top, respectively, and simultaneously rank R4 at the bottom. This reveals the result heterogeneity in a decision group, and in a sense necessitates the MCGDM for RSP. In addition, the second moment *σ*_*k*_, third moment *γ*_*k*_ and preferential priority *δ*_*i*_ are also reported in Table 3. It is noticed that the second moment of Shannon entropy approach (*σ*_1_ = 0.1081) is largest. This implies that Shannon entropy approach may have the most preferential difference among these robots and therefore could discriminate most decisively. Furthermore, the third moments of Shannon entropy approach and CRITIC approach are positive, and those of distance-based approach and ideal-point approach are negative. This reveals that distance-based approach and ideal-point approach have analogical skew-to-left preferential pat- terns, and would be uncomfortable if their relatively disagreeable robots are adopted, particularly for ideal-point approach with the largest skewness as *γ*_4_ = -0.5908. In addition, the preferential priority of R11 is largest (*δ*_11_ = 0.1638). This demonstrates that R11 is most preferred in ranking for the group. In consideration of the different rankings from different individual DMs, ranking R11 at the top seems to be a more probable compromise among different individual DMs.

**Table 3:** Robot performance results.

Robot	Shannon entropy	CRITIC	Distance	Ideal-point	Preferential priority *δ*_*i*_
R1	0.0947	0.0871	0.0986	**0.1040**	0.1571
R2	0.0766	0.0787	**0.1005**	0.1007	0.1439
R3	0.0680	0.0760	0.0897	0.0891	0.0593
R4	**0.0540**	**0.0677**	**0.0602**	**0.0539**	0.0269
R5	0.0661	0.0741	0.0786	0.0797	0.0344
R6	0.0741	0.0702	0.0664	0.0694	0.0320
R7	0.0924	0.0827	0.0796	0.0874	0.0557
R8	0.0797	0.0877	0.0851	0.0767	0.0559
R9	0.0675	0.0733	0.0789	0.0751	0.0331
R10	0.1039	0.0922	0.0834	0.0861	0.0786
R11	**0.1132**	0.1023	0.0875	0.0927	**0.1638**
R12	0.1098	**0.1081**	0.0916	0.0851	0.1592
Second moment *σ*_*k*_	**0.0191**	0.0127	0.0118	0.0137	
Third moment *γ*_*k*_	0.2673	0.7732	-0.5347	**-0.5908**	

In accordance with the derived values of second moment *σ*_*k*_, third moment *γ*_*k*_ and preferential priority *δ*_*i*_, the revised group decision matrix is obtained and presented in the following Table 4. In comparison with the original group decision matrix, majority of the performance results have been adjusted to reflect the individual preferences of individual DMs. Additionally, the discriminative performance results of robots using the MCGDM-IP are derived using the Eq (20) and presented in Table 4 as well, according to which a complete ranking among robots is easily obtained to support robot selection. Therefore, the rankings obtained from the proposed MCGDM-IP and individual DMs are compared in the following Table 5. Table 5 demonstrates that different individual DMs provide different rankings of robots. This is mainly originated from different weight determination rationales. Different weight elicitation methods, when the same input dataset is employed, generate different and potentially conflicting rankings.

**Table 4:** Revised group decision matrix.

Robot	Shannon entropy	CRITIC	Distance	Ideal-point	MCGDM-IP
R1	0.099421	0.090318	0.101491	0.107436	0.099667
R2	0.000124	0.000180	0.103371	0.104037	0.051928
R3	0.000107	0.000184	0.092362	0.092192	0.046211
R4	0.000070	0.000151	0.000039	0.000029	0.000072
R5	0.000104	0.000180	0.000067	0.000063	0.000103
R6	0.000131	0.000161	0.000048	0.000048	0.000097
R7	0.096814	0.000219	0.000067	0.090430	0.046883
R8	0.000148	0.090573	0.087679	0.000057	0.044614
R9	0.000108	0.000176	0.000068	0.000056	0.000102
R10	0.108729	0.095222	0.085961	0.089151	0.094766
R11	0.118419	0.105774	0.090165	0.095886	0.102561
R12	0.114961	0.111756	0.094376	0.088147	0.102310

**Table 5:** Ranking comparisons.

Robot	Entropy	CRITIC	Distance	Ideal-point	GDM-IP
R1	4	5	2	1	3
R2	7	7	1	2	5
R3	9	8	4	4	7
R4	12	12	12	12	12
R5	11	9	10	8	9
R6	8	11	11	11	11
R7	5	6	8	5	6
R8	6	4	6	9	8
R9	10	10	9	10	10
R10	3	3	7	6	4
R11	1	2	5	3	1
R12	2	1	3	7	2

In the following, the merits of the proposed MCGDM-IP are manifested in terms of analyzing the satisfaction index. As one of the most widely-employed method for real-life group decision problems, the simple additive weighting (SAW) method is known as a common aggregation method for group decisions [20]. In this regard, we calculate the satisfactory levels of each individual DM when using both SAW method and our MCGDM-IP, which are reported in Table 6 below. It is shown that the proposed MCGDM-IP could significantly increase the satisfactory levels of all decision makers.

**Table 6:** Satisfaction index.

	SAW	MCGDM-IP	Difference
Shannon entropy approach	0.9697	0.9828	+0.0131
CRITIC approach	0.9606	0.9998	+0.0392
Distance-based approach	0.9870	0.9998	+0.0128
Ideal-point approach	0.9822	0.9999	+0.0177
Decision group	0.9749	0.9956	+0.0207

In addition, the satisfactory level of the decision group is increased by $\frac{0.9956‐0.9749}{0.9749}\times 100\%=2.12\%$. Therefore, our MCGDM-IP is more satisfactory than the SAW method in solving the group decision problem. That is to say, the MCGDM-IP could give rise to a more satisfactory scheme to evaluate and select industrial robots.

Next, we compare the rankings of the proposed MCGDM-IP with previous DEA methods [32,33] and present in Table 7 below. It is apparent that the different methods produce different rankings among robots, which can effectively provide robot selection schemes. In general, the best and worst performers in the proposed MCGDM-IP are R11 and R4, respectively, while those in DEA methods are R12 and R6, respectively. Noteworthy that the most remarkable differences are associated with R4 and R10. The difference between the proposed MCGDM-IP and DEA methods are originated from distinct rationales for performance evaluation.

**Table 7:** Ranking comparisons with other methods.

Robot	Karsak	and	Ahiska	(2005)	Chu	et	al.	(2020)	MCGDM-IP
R1	8			9				3	
R2	6			6				5	
R3	5			4				6	
R4	4			5				12	
R5	3			2				9	
R6	12			12				11	
R7	7			8				7	
R8	11			10				8	
R9	10			7				10	
R10	9			11				4	
R11	2			3				1	
R12	1			1				2	

## 4. Conclusions

This study proposes a MCGDM method with individual preferences (MCGDM-IP) to effectively and rationally evaluate and select industrial robots. Shannon entropy approach, CRITIC approach, distance-based approach, and ideal-point approach, are employed to elicit criteria weights in an objective manner and therefore to represent individual DMs. The individual preferences from the aspects of both the preferential differences and preferential priorities for individual DMs’ assessments of alter- natives in a decision group are explored. Specifically, the preferential differences denote the preference degrees among different alternatives, and the preferential priorities represent the favorite ranking of the alternatives for each individual DM. A satisfaction index is proposed to manifest the merits of our MCGDM-IP. An illustrative example using the data drawn from the previous studies to indicate the effectiveness and validity of the proposed MCGDM-IP, in conjunction with the comparison with the results obtained from DEA models. The MCGDM-IP is capable of generating a complete ranking of robots for evaluation and selection, with the improvements of both individual and group satisfactory levels.

The novelties of this study are summarized as follows. First, each individual DM objectively derives the criteria weights based upon the evaluation dataset itself. This could effectively reduce the decision bias and to some extent enhance the decision quality. Second, the preferential differences among various robots and preferential priorities for each individual DM are analyzed to obtain a revised group decision matrix, and thereby to enhance the commitment among group members. Third, the satisfaction status of individual DMs and group are separately and comprehensively measured to demonstrate the feasibility and superiority of MCGDM-IP.

There exist several limitations that deserve further investigation in future research. First, only objective criteria weight elicitation approaches are utilized to denote individual DMs in our paper, for example, the TOPSIS and fuzzy TOPSIS [34]. In spite of the prominent advantages, the personal experience and subjective judgement of individual DMs are also significantly importance in decision process. Therefore, future research should develop subjective-objective integrated criteria weight elicitation approaches to denote the individual DMs. Second, the second and third moments in statistics are employed to describe the preferential differences in this study. Future research should make the adjustments using other meaningful and useful approaches. Third, the exponential distribution is used to adjust the decision matrix by integrating the second and third moments. Future research should untangle the feasibility of other distributions. Fourth, the deterministic situation is investigated in this study, with certain input data and preference. Future research should consider the uncertain circumstance with uncertain input data and preference [34,35].

## Supporting information

S1 File (XLSX)
